# Systemic and topical administration of spermidine accelerates skin wound healing

**DOI:** 10.1186/s12964-021-00717-y

**Published:** 2021-03-22

**Authors:** Daisuke Ito, Hiroyasu Ito, Takayasu Ideta, Ayumu Kanbe, Soranobu Ninomiya, Masahito Shimizu

**Affiliations:** 1 Department of Gastroenterology, Gifu University Graduate School of Medicine, Yanagido, Gifu City, 501-1194 Japan; 2 Department of Joint Research Laboratory of Clinical Medicine, Fujita Health University School of Medicine, 1-98 Dengakugakubo, Kutsukake-cho, Toyoake, Aichi City, 470-1192 Japan; 3 Department of Informative Clinical Medicine, Gifu University Graduate School of Medicine, Yanagido, Gifu City, 501-1194 Japan; 4 Department of Clinical Laboratory, Gifu University Hospital, Yanagido, Gifu City, 501-1194 Japan

**Keywords:** Spermidine (SPD), Wound healing, Urokinase-type plasminogen activator receptor (uPAR), Inflammation, Public health

## Abstract

**Background:**

The skin wound healing process is regulated by various cytokines, chemokines, and growth factors. Recent reports have demonstrated that spermine/spermidine (SPD) promote wound healing through urokinase-type plasminogen activator (uPA)/uPA receptor (uPAR) signaling in vitro. Here, we investigated whether the systemic and topical administration of SPD would accelerate the skin wound-repair process in vivo.

**Methods:**

A skin wound repair model was established using C57BL/6 J mice. SPD was mixed with white petrolatum for topical administration. For systemic administration, SPD mixed with drinking water was orally administered. Changes in wound size over time were calculated using digital photography.

**Results:**

Systemic and topical SPD treatment significantly accelerated skin wound healing. The administration of SPD promoted the uPA/uPAR pathway in wound sites. Moreover, topical treatment with SPD enhanced the expression of IL-6 and TNF-α in wound sites. Scratch and cell proliferation assays revealed that SPD administration accelerated scratch wound closure and cell proliferation in vitro.

**Conclusion:**

These results indicate that treatment with SPD promotes skin wound healing through activation of the uPA/uPAR pathway and induction of the inflammatory response in wound sites. The administration of SPD might contribute to new effective treatments to accelerate skin wound healing.

**Video Abstract**

**Supplementary Information:**

The online version contains supplementary material available at 10.1186/s12964-021-00717-y.

## Background

Skin wound healing is a complex process involving three phases: inflammation, cell proliferation, and tissue remodeling. In the inflammation phase, infiltrating neutrophils and macrophages play critical roles in the defense against bacterial infection and the resection of necrotic tissue [1]. A previous report demonstrated that the immediate induction of an inflammatory response after wounding is critical for the re-epithelialization of damaged skin [2, 3]. In the proliferation phase, fibroblasts and myofibroblasts interact and produce extracellular matrix, resulting in granulation tissue formation. The tissue remodeling process is primarily associated with tissue maturation and collagen degradation by matrix metalloproteinases (MMPs) derived from leukocytes and dermal fibroblasts [4].

Keratinocytes in the basal layer of the epidermis contact the basement membrane and proliferate. Upon epidermal wounding, keratinocytes at the wound edge undergo a transition from a nonmotile epithelial state to a mesenchymal-like state in which they lose cell–cell contacts and become motile. A previous study demonstrated that urokinase-type plasminogen activator (uPA) is upregulated at the wound edge where keratinocytes migrate and promotes cell migration [5]. In addition, u-PA upregulation and functional activity have been reported to significantly enhance endothelial cell viability, growth, and wound healing [6]. uPA plays a pivotal role in extracellular proteolysis and is thought to be critically involved in the modulation of angiogenesis via the interaction with its uPA receptor [7]. uPA receptor (uPAR) plays an important role in cell surface-associated plasminogen activation leading to disassembly and remodeling of the extracellular matrix [8]. uPAR expression is strongly enhanced during inflammation, immune responses, injury and stress and under conditions of tissue remodeling such as those during embryo implantation or wound healing [5, 9, 10]. The loss of uPAR function delayed the wound-healing response and impaired keratinocyte proliferation and migration [11].

Spermidine (SPD) is a naturally occurring polyamine, originally isolated from semen that is also found in cheese, corn, mushrooms, legumes, soya products and whole grains [12]. Polyamines are ubiquitous endogenous metabolites and essential organic compounds for cell growth and proliferation [13, 14]. A previous study reported that SPD is involved in cell proliferation and cell differentiation [15]. Moreover, polyamines are involved in a wide variety of cellular processes, as they participate in the regulation of gene expression through regulating enzyme activity, activating DNA synthesis, facilitating the interaction of DNA and protein, and protecting DNA molecules from putative damaging agents [16]. In wound models using human skin samples, levels of ornithine decarboxylase (ODC), the rate-limiting enzyme in polyamine metabolism, and adenosylmethionine decarboxylase 1 (AMD1), a polyamine regulator, rapidly increased at the wound edge [14]. In addition to SPD, spermine (SPM) is involved in wound healing because it rescued AMD1 knockdown and promoted keratinocyte migration and the re-epithelialization of human wounds ex vivo. Polyamines are essential for endothelial cell proliferation and angiogenesis, and it has been reported that the intracellular supply of ornithine for polyamine synthesis may play an important role in promoting placental angiogenesis and wound healing [17]. Although some studies have verified the effect of SPD on skin wound healing in vitro, the effect of SPD in vivo has remained unclear. Therefore, the present study investigated the effect of SPD on the wound-healing process in vivo.

## Material and methods

### Mice

C57BL/6 J mice (age 7–9 weeks, male) were obtained from Japan SLC Inc. (Shizuoka, Japan). All procedures were conducted in accordance with the guidelines of the National Institutes of Health Guide for the Care and Use of Laboratory Animals and the guidelines for the care and use of animals established by the Animal Care and Use Committee of Gifu University (Gifu, Japan).

### Reagents

SPD (> 99% purity) was obtained from Sigma-Aldrich (St Louis, MO). SPD (2 μg/wound) was added to 100 μl of white petrolatum. The formulation was heated to 60 °C and quickly mixed to emulsify the components. Distilled water containing SPD (5 mM) was systemically administered daily for 4 days before skin wound creation. MDI-2268 obtained from AOBIOUS INC (Gloucester, MA) was dissolved in 0.1% DMSO in lactated ringer buffer. After a skin wound had been created, mice received MDI-2268 (3 mg/kg) or vehicle by intraperitoneal administration for 2 days. Amiloride HCl (a uPA inhibitor) was obtained from Sigma-Aldrich (St Louis, MO). After a skin wound had been created, mice were intraperitoneally administered amiloride HCl (10 mg/kg) for every day.

### Establishment of a wound repair model and measurement of the wound area

A skin wound repair model was established as shown in our previous study [18]. In brief, mice were anesthetized, and their backs were shaved and sterilized with 70% ethanol. Two excised wounds were created using a 5 mm sterile circular punch (Kai Industries Co., Gifu, Japan) from the right- and left-upper paravertebral regions of the mouse, and the entire skin thickness was removed. The biopsy sites were coated with 100 μl of white petrolatum containing 2 μl of SPD and 2 μl of PBS, or 4 μl of PBS as a control, on days 0 and 2. The mice were wrapped with a tight-fitting bandage to protect the biopsy sites. Wounds were checked and photographed every other day. The wound area was measured at the indicated time points with a ImageJ software (version 1.37; NIH, Bethesda, MD), and the results are expressed as the percentage closure relative to original size (1 − [wound area]/[original wound area] × 100). Suggestive signs of topical infection were not detected in the wound area. Each treatment was tested, and the results from minimum of 4 independent animals/group were averaged.

### Extraction of RNA and quantitative RT-PCR

Tissues from the biopsy site were excised 0, 24, 48 h after wound creation. Wound site tissues taken from the 2–3 mm surrounding the wound edge were immediately frozen after collection. Total RNA was extracted from the wound site using ISOGEN II reagent (Nippon Gene, Tokyo, Japan), and first-strand cDNA was synthesized using the High Capacity cDNA Reverse Transcription Kit (Applied Biosystems, Foster City, CA). Quantitative real-time RT-PCR was performed using specific primer–probe sets to amplify VEGF mRNA with TaqMan^®^ Gene Expression Assays and Universal PCR Master Mix (Applied Biosystems) or to amplify IL-6, TNF-α, MMP-2, MMP-9 and EGF mRNA with QuantiTect SYBR Green PCR Master Mix (Qiagen GmbH, Hilden, Germany). Each sample was analyzed on a Light-Cycler^®^ 480 system (Roche Diagnostic Systems, Basel, Switzerland). The expression level of each gene was normalized against that of GAPDH mRNA. The primer sequences used for qRT-PCR were as follows: IL-6-fwd, TCCAGTTGCCTTCTTGGGAC; IL-6-rev, GTACTCCAGAAGACCAGAGG; TNF-α-fwd, CACAGAAAGCATGATCCGCGACGT; TNF-α -rev, CGGCAGAGAGGAGGTTGACTTTCT; MMP-2-fwd, CCCCTGATGTCCAGCAAGTAGA; MMP-2-rev, AGTCTGCGATGAGCTTAGGGAAA; MMP-9-fwd, CCCTGGAACTCACACGACATCTTC; MMP-9-rev, GGTCCACCTTGTTCACCTCATTTT; EGF-fwd, ATGGGAAACAATGTCACGAAC; EGF-rev, TGTATTCCGTCTCCTTGGTTC; GAPDH-fwd, TGCACCACCAACTGCTTAG; and GAPDH-rev, GGATGCAGGGATGATGTTC.

### Western blot analysis

Skin tissues taken from approximately 2–3 mm surrounding the wound edge were homogenized in CelLytic MT Cell Lysis Reagent (C3228, Sigma-Aldrich). Proteins were separated from the lysate by sodium dodecyl sulfate–polyacrylamide gel electrophoresis (SDS-PAGE) and transferred to a nitrocellulose membrane. After being blocked with 5% skim milk and 1% bovine serum albumin in Tris-buffered saline-Tween at room temperature for 1 h, the membrane was incubated with rabbit anti- PLAUR (Bioss Antibodies, bs-1927R, 1:1,000), rabbit anti-PCNA (Cell Signaling, D3H8P/#13110, 1:1,000) and anti-GAPDH (Cell Signaling Technology) primary antibodies for 60 min and then incubated with peroxidase labeled anti-rabbit IgG antibody (Santa Cruz Biotechnology) for 60 min at room temperature. Detection of protein bands was performed with ECL Plus reagent (GE Healthcare UK Ltd., England).

### Enzyme-linked immunosorbent assay (ELISA)

Blood was collected before and after skin wound creation, and serum was collected by centrifugation. Serum was used to measure uPA protein levels with a Mouse uPA ELISA Kit (Abcam, Cambridge, MA, USA) according to the manufacturer’s instructions.

### Scratch assay

A scratch assay was carried out as in our previous study [19]. In brief, embryos were harvested at embryonic day 12.5 to establish mouse embryonic fibroblasts (MEFs). MEF cultures were prepared using standard techniques [20]. Cells were maintained in complete RPMI1640 (FUJIFILM Wako Pure Chemical Corporation, Osaka, Japan) medium supplemented with 10% fetal bovine serum, penicillin/streptomycin, and l-glutamine (Gibco^®^, Invitrogen, Life Technologies, Grand Island, NY). Cultured MEFs from mice were grown in 12-well plates. When the cells reached confluence, a scratch was made across the cell monolayer with a yellow pipette tip (approximately 0.5 mm in width). After scratching, the cells were washed twice with PBS and SPD (4 μM, 20 μM and 100 μM) was then immediately added to the serum-free culture medium (SFM; RPMI-1640). The culture medium was removed at 24 and 48 h after scratching, and the cells were immersed in 4% paraformaldehyde for 30 min for immobilization. The cells were then stained with crystal violet for 1 h, and three representative scratched areas for each experimental condition were photographed. Changes in the non-wound closure area were measured using ImageJ software.

### Cell viability and cytotoxicity assays

The cell viability of the cultured cells was quantified using the Cell Counting Kit (CCK) -8 assay (Dojindo Molecular Technologies, Kumamoto, Japan) and an iMark™ microplate reader (BIO-RAD, Hercules, CA), according to the manufacturer’s instructions. After the cells were confluent, the medium was changed to SFM, SPD was added, and the cells were cultured for 24 h. The cell viability data are presented as a percent compared to control cells cultured in parallel in medium only.

### Statistical analyses

Values are expressed as the means ± standard errors of the mean (SEMs). The statistical significance of differences in the wound-healing rate were assessed using one-way repeated measures analysis of variance (ANOVA). Comparisons between the experimental groups were analyzed with the Kruskal–Wallis test followed by Scheffe’s F-test. Significance was established at *p* < 0.05.

## Results

### Topical and systemic treatment with SPD promoted skin wound healing in mice

**Fig. 1:**
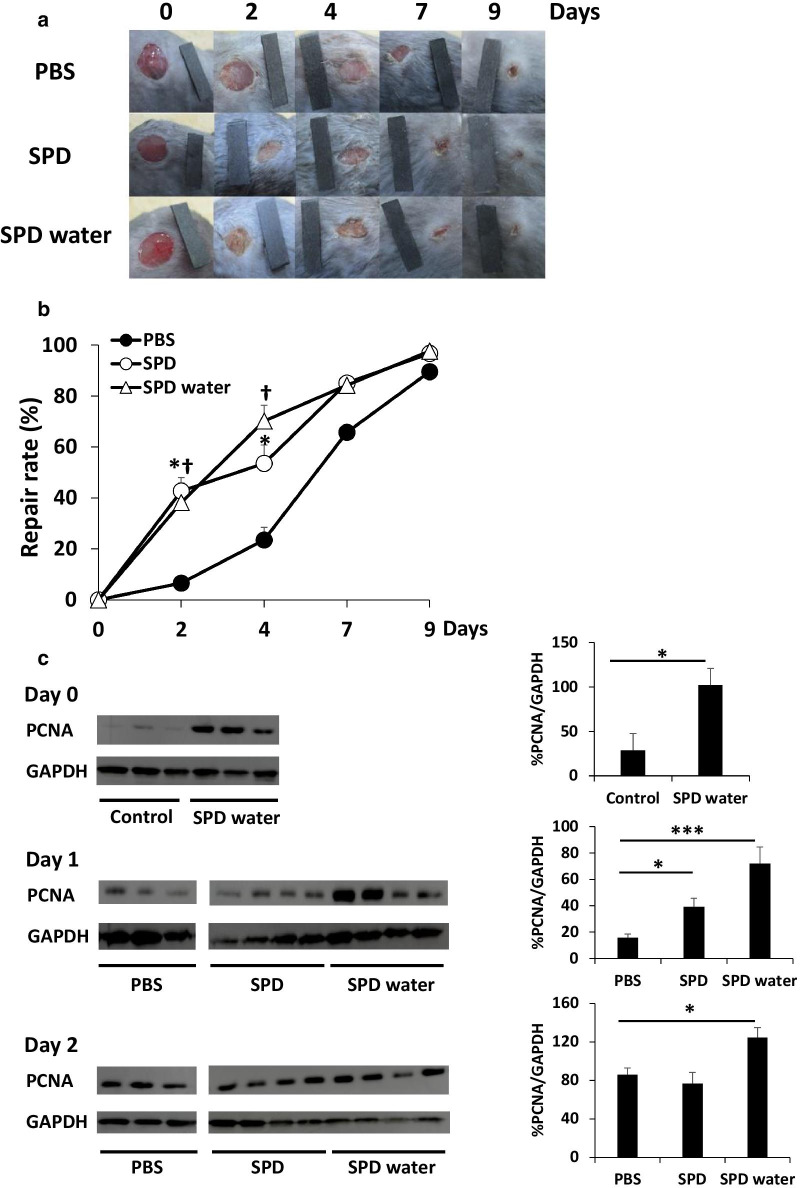
Acceleration of skin wound healing by the systemic and topical administration of SPD. **a**, **b** Two independent excisional biopsies (5 mm in diameter) were taken from the right and left dorsal side of each mouse (n = 6). Distilled water containing spermidine (5 mM) was administered daily from 4 days before skin wound creation. Biopsy sites were treated with white petrolatum containing PBS or SPD (2 μg/wound) 0 and 2 days after skin wound creation. Each wound was recorded via digital photography, and the scale bar represents 10 mm. The mean rate of repair was calculated based on the original wound area of each biopsy site. *p < 0.05, white petrolatum with SPD group compared to white petrolatum with PBS group. ^†^*p* < 0.05, SPD-containing water + white petrolatum with PBS group compared to white petrolatum with PBS group. **c** PCNA protein levels after wound creation were examined by western blot analysis and normalized using GAPDH protein levels. Each data point and error bar represent the mean and SE, respectively, of data from triplicate or quadruplicate samples. *Indicates a statistically significant differences; *p* < 0.05. ***Indicates a statistically significant difference; *p* < 0.005

We first examined the effect of topical and systemic administration of SPD on skin wound healing in vivo. Two identical full-thickness skin biopsies were taken from the right and left subscapular regions of individual mice. The mice were divided into the following three groups: the untreated group, topical SPD administration group, and systemic SPD administration group. The skin wounds were observed every other day, and alterations in wound size over time were calculated using digital photography. Both topical and systemic administration of SPD significantly accelerated wound healing at 2 and 4 days after wound creation (Fig. 1a, b). Indeed, 50% wound closure was achieved after 5.3 ± 0.2 days at sites treated with PBS, 3.7 ± 0.8 days at sites topically treated with SPD, and 2.8 ± 0.5 days at sites systemically treated with SPD (Fig. 1b). Next, the expression of PCNA at the wound site was measured using western blot analysis. Topical SPD treatment significantly increased PCNA expression at 1 day after skin wound creation. The systemic administration of SPD enhanced PCNA expression in the skin tissues before skin wound creation, and PCNA expression in the wound site of mice in the systemic SPD administration group was also increased (Fig. 1c). These results indicate that the topical and systemic administration of SPD accelerated skin wound healing and induced cell proliferation at the wound site in the mice.

### Administration of SPD increased uPA and uPAR expression, and induced uPAR cleavage

**Fig. 2:**
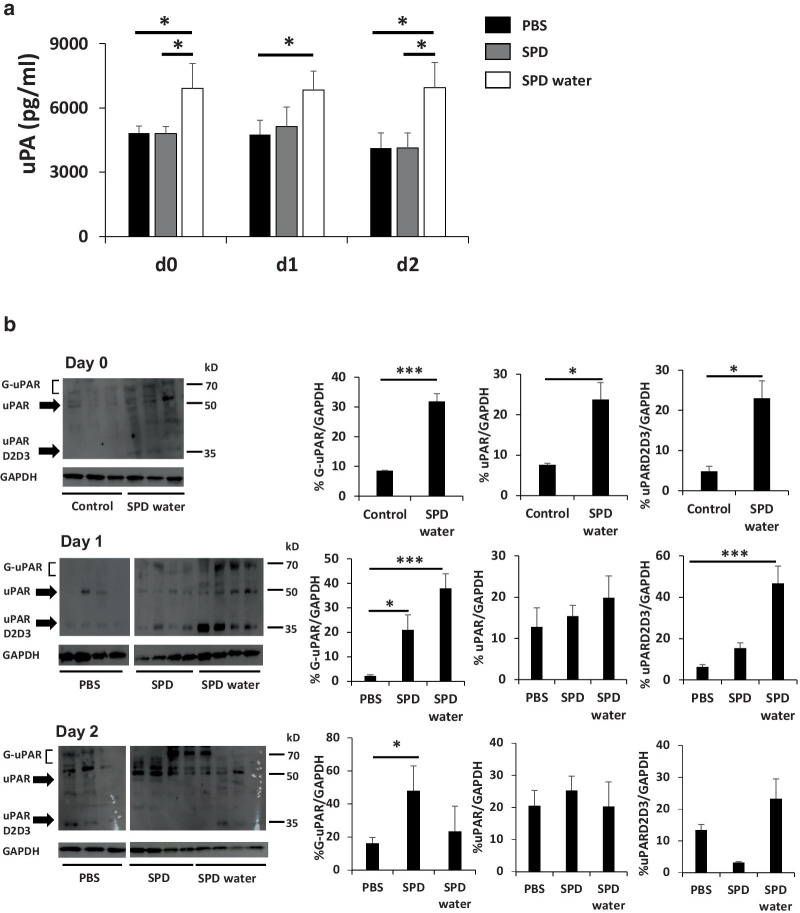
The systemic and topical administration of SPD enhances G-uPAR production and uPAR cleavage. **a** The serum uPA concentration in the mice treated with systemic or topical SPD was measured using ELISA (n = 4 mice). **b**–**d** The expression of G-uPAR, uPAR, uPARD2D3, and GAPDH in the wound sites was measured using western blot analysis. Skin tissue lysates (20 µg/protein) were used for the experiment and analyzed by immunoblotting. Chemiluminescent signals were quantified using ImageJ software and normalized to GAPDH signals. *Indicates a statistically significant differences; *p* < 0.05. ***Indicates a statistically significant difference; *p* < 0.005

uPA and uPAR play a central role in cell surface-associated plasminogen activation leading to degradation and remodeling of the extracellular matrix [21]. The loss of uPAR function in uPAR-knockout mice delayed skin wound repair, and impaired keratinocyte proliferation and migration [11]. Therefore, serum uPA protein levels were measured in mice treated with SPD using ELISA before and after skin wound creation. Systemic SPD administration significantly increased the serum uPA level before skin wound creation (Fig. 2a). In addition, western blot analysis revealed that systemic SPD administration alone significantly increased the expression of uPARD2D3 (approximately 37 kDa) (Fig. 2b). Moreover, systemic administration of SPD increased the expression of glycosylated uPAR (G-uPAR) (approximately 55–70 kDa) and uPARD2D3 in the wound site (Fig. 2b). Similarly, topical SPD treatment significantly increased the expression of G-uPAR at the wound sites (Fig. 2b).

### uPA-uPAR signaling regulates the effect of spermidine on skin wound healing

**Fig. 3:**
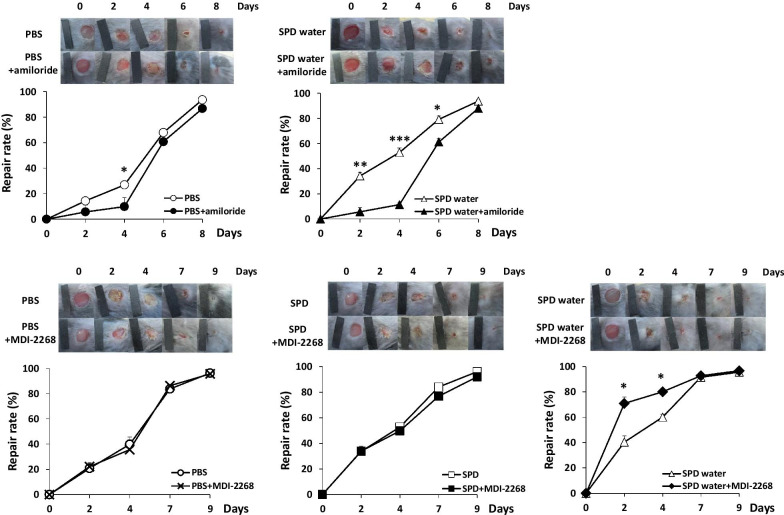
Regulation of uPA activity affected the acceleration of skin wound healing induced by SPD treatment. **a**–**e** Multiple independent excisional biopsies (5 mm diameter) were taken from the right and left dorsum of each mouse. **a**, **b** To inhibit uPA activity, amiloride (10 mg/kg) was intraperitoneally administered to the mice every day after wound creation. The rates of wound closure among the following four groups were compared: the SPD untreated group, SPD untreated + amiloride group, SPD systemic administration group and SPD systemic administration + amiloride group. **c**–**e** To inhibit PAI-1 function, MDI-2268 was administered intraperitoneally (3 mg/kg) to mice for 2 consecutive days after wounding. The rates of wound closure among the following six groups were compared: the SPD untreated group, SPD untreated + MDI-2268 group, SPD topical administration group, SPD topical administration + MDI-2268 group, SPD systemic administration group, and SPD systemic administration + MDI-2268 group. The mean rate of wound repair was calculated based on the original wound area of each biopsy site. *Indicates a statistically significant differences; *p* < 0.05. **Indicates a statistically significant difference; *p* < 0.01. ***Indicates a statistically significant difference; *p* < 0.005

A previous study demonstrated that amiloride inhibited the uPA-uPAR pathway in a mouse lung tumor model [22]. Next, to determine whether the uPA-uPAR pathway contributes to the ability of SPD to promote skin wound healing, we intraperitoneally administered amiloride (10 mg/kg) daily. In the mice that drank water without SPD, wound healing was significantly impaired by the administration of amiloride (Fig. 3a). In the mice that drank water with SPD, wound healing was also impaired by the administration of amiloride 2–6 days after wound creation (Fig. 3b). These results suggest that the uPA-uPAR pathway is involved in the effect of SPD administration in promoting wound healing. In fact, 50% wound closure was achieved after 4.4 ± 0.2 days at sites with PBS, 4.9 ± 0.8 days at sites systemically treated with amiloride alone, 3.8 ± 0.3 days at sites systemically treated with SPD, and 4.9 ± 0.2 days at sites systemically treated with SPD and amiloride (Fig. 3a, b).

A previous study demonstrated that PAI-1 inhibited uPA activation in macrophages [23]. Next, we examined the effect of PAI-1 on skin wound healing using MDI-2268 which is a PAI-1 inhibitor. The skin wound repair rate in the mice systemically administered SPD was significantly increased by the administration of MDI-2268 (Fig. 3c–e). However, the administration of MDI-2268 did not affect the skin wound repair rate in the mice topically administered with and without SPD. Indeed, 50% wound closure was achieved after 4.2 ± 0.3 days at sites treated with PBS, 4.1 ± 0.2 days at sites treated with MDI-2268 alone, 3.3 ± 0.7 days at sites treated with systemic SPD, 1.4 ± 0.2 days at sites treated with systemic SPD and MDI-2268, 3.8 ± 0.3 days at sites treated with topical SPD and 4.6 ± 0.6 days at sites treated with topical SPD and MDI-2268 (Fig. 3c–e).

### Effects of SPD administration on the expression of pro-inflammatory cytokines, MMPs, and growth factors

**Fig. 4:**
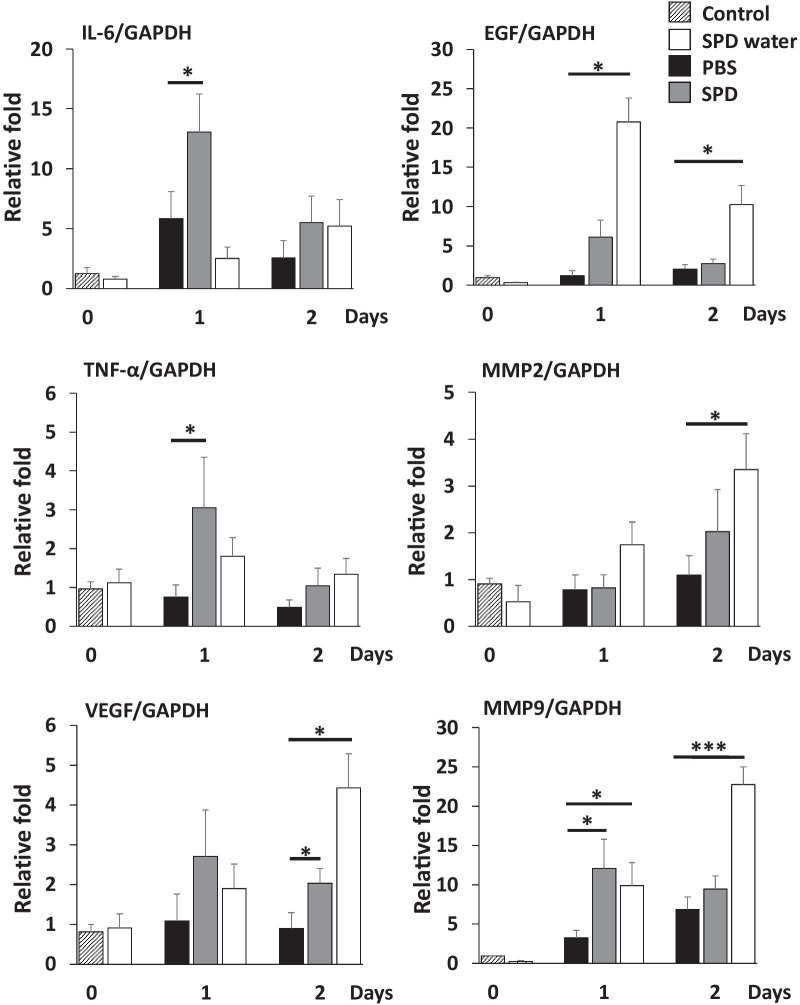
The mRNA expression of proinflammatory cytokines, growth factors, and MMPs at the wound sites of SPD-treated mice. The relative mRNA expression of IL-6, TNF-α, VEGF, EGF, MMP2 and MMP-9 at 0, 24, and 48 h after wound creation was measured using quantitative RT-PCR (n = 4). The results were normalized to the mRNA expression of GAPDH. *Indicates a statistically significant difference; *p* < 0.05. ***Indicates a statistically significant difference; *p* < 0.005

The first phase in the skin wound healing process is the inflammatory phase, in which various pro-inflammatory cytokines are upregulated at the wound site. A previous report demonstrated that pro-inflammatory cytokines are directly and/or indirectly involved in the wound healing process, and their upregulation was required for optimal skin wound healing [24]. Therefore, we evaluated the mRNA expression of the pro-inflammatory cytokines IL6 and TNF-α in the wound site after treatment with SPD. As shown in Fig. 4, the expression of IL-6 and TNF-α in the skin tissues at 24 h after wound creation was significantly enhanced in mice topically treated with SPD. In contrast, the systemic administration of SPD did not affect the expression of these cytokines after wound creation. Previous studies demonstrated that growth factors such as VEGF and EGF, and MMPs are involved in optimal skin wound healing [25, 26]. We examined the expression of VEGF, EGF, and MMPs in the wound site after SPD treatment (Fig. 4). The expression of EGF in the wound site was significantly increased after the systemic administration of SPD. VEGF expression was enhanced by the topical and systemic administration of SPD. Moreover, MMP expression was also upregulated by SPD administration.

### The addition of SPD accelerated scratch wound closure and cell proliferation

**Fig. 5:**
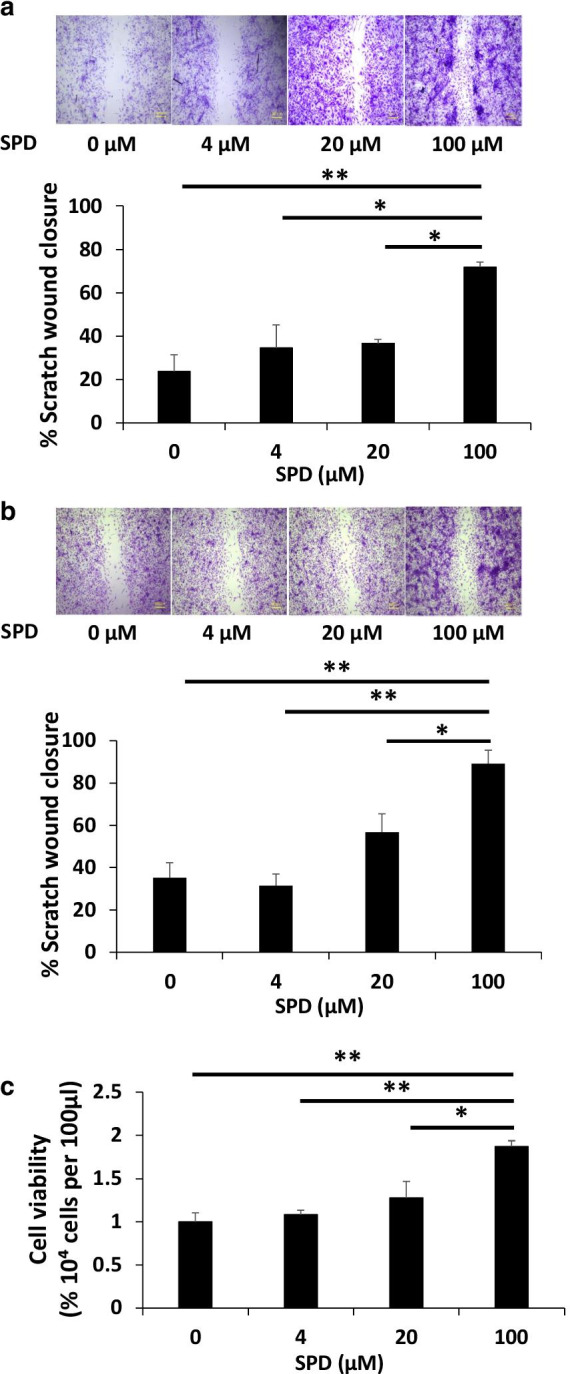
The addition of SPD accelerated scratch wound closure and cell proliferation in vitro. MEFs were cultured in 12-well plates. When the cells reached confluence, the monolayer was injured by scratching across the plate with a yellow pipette tip (approximately 0.5 mm in width). After scratching, the cells were washed twice with PBS, after which SPD was added. **a**, **b** Digital photographic images taken at 24 h (**a**) and 48 h (**b**) after scratching. The rate of scratch wounds closure was determined using ImageJ software. Each data point and error bar represent the mean and SEM, respectively. **c** The cell viability of MEFs after the administration of SPD was quantified using the CCK-8 assay. *Indicates a statistically significant difference; *p* < 0.05. **Indicates a statistically significant difference; *p* < 0.01

To evaluate the effect of SPD on wound healing in vitro, we prepared MEFs and conducted scratch wound assays using these cells. As shown in Fig. 5a, b, the rate of scratch wound closure of MEFs in medium to which SPD was added was significantly increased compared with that in MEFs in medium without SPD. Moreover, the rate of scratch wound closure increased in a concentration-dependent manner. Next, a cell proliferation assay was performed to examine the effects of SPD at several concentration on MEF proliferation in serum-free medium. MEF proliferation was increased in a concentration-dependent manner upon treatment with SPD at concentrations close to that used for the scratch assay (Fig. 5c). These results indicated that SPD is involved in promoting wound healing by upregulating cell proliferation and migration.

## Discussion

In the current study, we demonstrated that systemic and topical SPD treatment significantly accelerated skin wound healing (Fig. 1a, b). We found that SPD treatment promoted skin wound healing through the uPA/uPAR pathway (Figs. 2, 3). Moreover, topical SPD treatment induced an earlier increase in inflammatory cytokine expression after skin wound creation (Fig. 4). This enhanced inflammatory cytokine expression after the topical administration of SPD might be involved in the acceleration of wound healing.

Present in all higher eukaryotic cells, polyamines are essential for cell growth and differentiation [27]. In addition, when putrescine is systemically administered, it is mostly converted to other non-polyamine metabolites in the intestine by the enzyme diamine oxidase [28]. However, SPD is not enzymatically degraded in the alimentary tract [28]. Most intracellular polyamines exist as a polyamine‐RNA complex and play important roles in transcription and translation in the cell cycle through causing a structural change in RNA, especially in the transitions from G_1_ phase to S phase and from G_2_ phase to M phase [29]. In the present study, PCNA expression was significantly enhanced by systemic and topical treatment with SPD in vivo (Fig. 1c). Furthermore, the proliferation assay revealed that the number of MEFs approximately doubled after 24 h in the presence of SPD (Fig. 5c). In addition, the rate of scratch wound closure was 80% in SPD-high containing medium (Fig. 5a, b). These results indicated that the enhancement of cell proliferation was partially involved in the acceleration of scratch wound closure and SPD also increased invasive capacity.

uPAR regulates proteolysis by binding uPA and activating many intracellular signaling pathways [30]. Coordination of extracellular matrix (ECM) protein degradation with MMPs induced by uPAR promotes cell migration, proliferation, and survival. G-uPAR is a highly glycosylated, glycosylphosphatidylinositol-anchored receptor with three extracellular domains (D1–D3). Proteolytic cleavage can occur in the linker region between D1 and D2 yielding the fragment uPARD2D3, which can undergo further cleavage of the glycosylphosphatidylinositol linker releasing the soluble form uPARD2D3 [31]. Several studies have indicated that uPARD2D3 is involved in cell signaling and stem cell mobilization [32, 33]. Upregulation of G-uPAR and uPARD2D3 was also found to be involved in skin wound healing [34]. Therefore, the present study examined the effect of SPD administration on uPA-uPAR activation. The results showed that the topical administration of SPD increased the expression of G-uPAR, and the systemic administration of SPD upregulated both G-uPAR and uPARD2D3 (Fig. 2b–d). uPAR binds pro-uPA or uPA, and bound uPA promotes the cleavage of plasminogen. Cleaved plasminogen actives plasmin, which promotes the cleavage of pro-uPA. Thus, plasmin and uPA which can activate each other, form a positive feedback loop [35]. Plasmin cleaves and activates MMPs, which contributes to uPAR cleavage, ECM degradation, and growth factor activation [36].

Amiloride is a moderately potent inhibitor of uPA that does not inhibit tissue-type plasminogen activator or other serine proteases, such as kallikrein, thrombin, or plasmin [37]. The effect of SPD promoting wound healing was inhibited by amiloride administration (Fig. 3b). The administration of amiloride also inhibited the normal wound-healing process (Fig. 3a). These results revealed that uPA is critical for optimal wound healing and the accelerated wound healing observed after the administration of SPD. Recently, a novel inhibitor (MDI-2268) against PAI-1, which impairs the activation of uPA-uPAR signaling, was developed and proven effective in a mouse model of deep vein thrombosis [38]. In the present study, skin wound healing in systemic SPD-treated mice was significantly promoted by the administration of MDI-2268 (Fig. 3e). In contrast, treatment with MDI-2268 did not affect skin wound repair in untreated and topical SPD-treated mice (Fig. 3c, d). The dependence of SPD-induced acceleration of wound healing on uPA-PAR signaling was more pronounced in systemic SPD-treated mice than in topical SPD-treated mice because the expression of uPA, G-uPAR, and uPARD2D3 in systemic SPD-treated mice was upregulated to greater extent. Therefore, the effect of MDI-2268 might have been enhanced in systemic SPD-treated mice.

Wound healing is a complex event that includes homeostasis, inflammation, granulation by cell proliferation, matrix deposition, and tissue remodeling. These phases depend on the interactions of cytokines, growth factors, chemokines, and chemical mediators with regulatory functions from various cells [39]. Previous studies have shown that SPD has anti-inflammatory effects [40]. However, as far as we know, there have been no reports confirming an inflammatory state when SPD is used to treat skin wound sites in vivo. Previous studies demonstrated that the excessive polyamines increased oxidative stress [41, 42]. In the present study, the topical administration of SPD significantly upregulated the expression of IL-6 and TNF-α in the wound site (Fig. 4). The increase in pro-inflammatory cytokines may be due to increased oxidative stress in these wound sites [43]. Previous studies have reported that IL-6 deficiency impairs wound healing via the inhibition of keratinocyte proliferation [44], and TNF-α upregulation has been found to increase keratinocyte growth factor production after wound formation [45]. These results suggest that the topical administration of SPD after wound creation induces mild oxidative stress and increases inflammatory cytokines.

## Conclusion

In summary, the systemic and topical administration of SPD accelerated skin wound healing via increases in G-uPAR and uPARD2D3. These increases in G-uPAR and uPARD2D3 induced cell proliferation and migration in vivo and in vitro. Moreover, the topical administration of SPD induced pro-inflammatory cytokine production, and the increase in these cytokines in wound sites may be involved in the acceleration of wound closure. Thus, the administration of SPD may constitute an attractive strategy to accelerate skin wound healing.

## Data Availability

The datasets used and/or analyzed during the current study are available on reasonable request.

## Abbreviations

SPD: Spermidine
SPM: Spermine
uPA: Urokinase-type plasminogen activator
uPAR: UPA receptor
G-uPAR: Glycosylated uPAR
ODC: Ornithine decarboxylase
AMD1: Adenosylmethionine decarboxylase 1
IL-6: Interleukin 6
TNF-α: Tumor necrosis factor-alpha
MMP: Mitochondrial membrane potential
VEGF: Vascular endothelial growth factor
EGF: Epidermal growth factor
PCNA: Proliferating cell nuclear antigen
GAPDH: Glyceraldehyde-3-phosphate dehydrogenase
PAI: Plasminogen activation inhibitor
VAP-1: Vascular adhesion protein-1
ECM: Extracellular matrix
MEF: Mouse embryonic fibroblasts
PBS: Phosphate buffered saline
SFM: Serum-free culture medium
SDA-PAGE: Sodium dodecyl sulfate polyacrylamide gel electrophoresis
ELISA: Enzyme-linked immunosorbent assay
CCK: Cell counting kit
ANOVA: Analysis of variance
SEM: Standard errors of the mean
